# Novel Human Induced Pluripotent Stem Cell-Based Model for Retinal Pigment Epithelial Cells to Reveal Possible Disease Mechanisms for Macular Degeneration in Pseudoxanthoma Elasticum

**DOI:** 10.1155/2024/6939920

**Published:** 2024-09-21

**Authors:** Taina Viheriälä, Heidi Hongisto, Lyydia Saari, Marika Oksanen, Tanja Ilmarinen, Suvi Väärämäki, Hannu Uusitalo, Pasi Nevalainen, Heli Skottman

**Affiliations:** ^1^ Faculty of Medicine and Health Technology Tampere University, Tampere, Finland; ^2^ Centre for Vascular Surgery and Interventional Radiology Tampere University Hospital and Tampere University, Tampere, Finland; ^3^ SILK Department of Ophthalmology Faculty of Medicine and Health Technology Tampere University, Tampere, Finland; ^4^ Tays Eye Centre Tampere University Hospital, Tampere, Finland; ^5^ Department of Internal Medicine Tampere University Hospital, Tampere, Finland

## Abstract

Pseudoxanthoma elasticum (PXE) is a rare metabolic disease with autosomal recessive inheritance. The manifestation in PXE is represented by retinal complications, pseudoxanthomas of the skin folding areas, and arterial calcification. The retinal complications are caused by the calcification of Bruch's membrane beneath retinal pigment epithelial cells (RPE) that can lead to retinal macular degeneration. The exact mechanism for the retinal pathophysiology is not known, and patients have variable symptoms and findings. Two induced pluripotent stem cell (hiPSC) lines from a patient carrying the common homozygous mutation c.3421C > T, p.Arg1141X in the ATP-binding cassette transporter gene (*ABCC6*; OMIM264800) were established and fully characterized. Then, RPE cells were differentiated, and molecular and functional characterization was conducted as a comparison to healthy controls. Data demonstrated that PXE-specific high-quality hiPSC lines can be established from a skin biopsy regardless of the skin-related disease phenotype and disease-specific RPE differentiation is feasible. The molecular and functional assessment of the PXE-specific RPE indicated increased pigmentation and reduced epithelial barrier functions as well as phagocytosis activity as compared to healthy controls. Although preliminary data, this indicates possible RPE-dependent factors that might explain the individual vulnerability of the retinas for macular degeneration in PXE. Future validation of the novel findings with additional PXE patients will be important.

## 1. Introduction

Pseudoxanthoma elasticum (PXE) is a rare, currently incurable, metabolic disease with autosomal recessive inheritance. The incidence has been estimated to be 1 : 25,000–100,000 [1]. PXE is caused by biallelic mutations in the adenosine triphosphate (ATP)-binding cassette transporter 6 (*ABCC6*) gene on chromosome 16 encoding the ABCC6 protein. The mutations in the gene lead to inefficient cellular ATP transport from the cell in the liver and further to low levels of plasma pyrophosphate (PPi) concentration, associated with progressive and extensive soft tissue calcification that leads to elastic fiber fragmentation in various soft conjunctival tissues [2, 3]. The manifestation of PXE is represented by retinal complications, papular skin change, large skin folds, and cardiovascular problems. The retinal complications are caused by the progressive calcification of the Bruch's membrane (BM), the pentalaminar membrane that is developed during embryogenesis between retinal pigment epithelium (RPE) and choroid and constitutes an anatomic barrier for the interchange between the choriocapillaris and RPE. The BM calcification and complications have a progressive nature and without known cure can lead ultimately to retinal macular degeneration [4–6].

The exact mechanism for the retinal pathophysiology is not known, but the progressive calcification of BM typically makes it brittle causing breaks and crack-like dehiscence known as angioid streaks. The extensive calcification will eventually lead to significant visual morbidity at a relatively young age due to choroidal neovascularization (CNV) and macular atrophy [7]. Although the progression of wet macular degeneration can be ameliorated by vascular endothelial growth factor (VEGF) inhibitors [8], no interventions are demonstrating the effect on the progression of calcification. Previously, it has been found that the reflectivity profiles measured with OCT from relatively young patients with PXE are different from controls and that the RPE-BM peak reflectivity performs well in discriminating patients with PXE from controls [4]. A recent study also showed reduced quantitative autofluorescence in PXE, which suggests reduced lipofuscin levels within the RPE [9]. These levels were associated with the extent of calcification, implicating that BM calcification in PXE affects the vitality of the outer retina. Another previous study has also proposed that the diffusion properties of the BM may be compromised and thus, vitamin A delivery to the outer retina is reduced. This hypothesis was underscored by a pathophysiological study demonstrating impaired rod-mediated dark adaptation in eyes with PXE as well as improvement of dark adaptation kinetics following vitamin A supplementation [10]. They also concluded later that the pathologic characteristics of this BM disease may be dominated by rod photoreceptor degeneration and/or dysfunction and that the putative RPE-photoreceptor separation may further impair rod function, while inner retinal abnormalities appear to be correlated with overall dysfunction [11]. Thus, the BM calcification may be the primary cause of the mutation, and only as a secondary cause of the RPE functionality being compromised and further leading to neural retina degeneration. However, a detailed assessment of the retinal changes has not been performed and the disease mechanism remains unknown. For this, patient-specific*in vitro* disease models could bring novel solutions.

In this study, we aimed to generate human induced pluripotent stem cell (hiPSC) lines from a PXE patient carrying heterozygous mutation c.3421C > T, p.Arg1141X in the *ABCC6* gene to establish an efficient in vitro disease model that can be used for studying disease mechanism specifically in patient-derived cells. Our primary hypothesis was that the hiPSC reprogramming could be possible from the patient's skin fibroblast regardless of the disease phenotype in the skin. Our secondary hypothesis was that if the RPE cells can be differentiated from the PXE-specific hiPSCs, their phenotype based on molecular and functional characterization reveals no differences as compared to the control cells due to the BM-specific nature of the PXE without any known association of RPE-related pathophysiology. As a conclusion, we were able to establish two high-quality hiPSC lines with pluripotency characteristics and efficient RPE differentiation was feasible using both of the cell lines. However, the molecular and functional assessment of PXE-specific hiPSC-RPE cells revealed interesting differences as compared to healthy controls providing important future research targets in understanding the retinal-specific disease mechanisms of PXE.

## 2. Materials and Methods

### 2.1. hiPSC Establishment and Characterization

Tampere University, Faculty of Medicine and Health Technology, has the approval of the National Authority Fimea (Dnro FIMEA/2020/003758) to conduct research on human embryos. The research group has supportive statements from the Regional Ethics Committee of the Expert Responsibility area of Tampere University Hospital to derive, culture, and differentiate human embryonic stem cell (hESC) lines (R05116) and to establish and use hiPSC lines in ophthalmic research (R16116). No new hESC lines were derived for the research conducted here and only previously established hESC line Regea08/017 was used as one of the control cell lines. Here, the establishment of the two hiPSC lines from a PXE patient (PXE006.TAU.f.D and PXE006.TAU.f.E) and his nearly age- (66-year-old male) and gender-matched healthy control without vision-related problems (WT007.TAU.f.A) are reported. The selected 59-year-old male patient had end-stage complications of PXE such as severely decreased visual acuity and critical ischemia of the right lower extremity in addition to type 2 diabetes, hypercholesterolemia, and arterial hypertension.

The primary skin fibroblasts from skin biopsy samples were expanded in fibroblast culture medium (IMDM with 10% FBS, 2 mM L-glutamine, and Pen/Strep, all from Life Technologies). The hiPSC lines were generated by reprogramming fibroblasts using a Sendai virus-based vector delivering the Yamanaka factors OCT4, SOX2, KLF4, and c-MYC (CytoTune-iPS 2.0 Sendai Reprogramming Kit, Thermo Fisher Scientific). Human Mitomycin C-treated foreskin fibroblasts (ATCC-2429) cultured in KnockOut™ Dulbecco's modified Eagle's medium (KO-DMEM) containing 20% KO-SR, 2 mM GlutaMAX, 1% MEM nonessential amino acids, 0.1 mM 2-mercaptoethanol, 50 U/mL penicillin-streptomycin (all from Gibco, Thermo Fisher Scientific), and 8 ng bFGF/ml (PeproTech Inc., Rocky Hill, NJ) were used as feeder cells for generated hiPSC.

After the manual selection of two morphologically high-quality hiPSC colonies from the PXE patient fibroblasts and one from the healthy control fibroblasts, the removal of the reprogramming vectors was confirmed with quantitative RT-PCR. The further expansion of the hiPSCs (from passage 4 onwards) as individual lines and cryopreservation were conducted to establish adequate master and working cell banks for research purposes. For that, feeder-free culture method was used as previously described [12] on Corning® CellBIND® 24-well culture plates (Corning, Corning, New York, NY, USA) coated with human recombinant laminin-521 (LN521™, 0.75 *μ*g/cm^2^, Biolamina, Sundbyberg, Sweden) and using Essential 8™ Flex Medium (E8, Thermo Fisher Scientific) supplemented with Essential 8 Flex supplement (1x) and 50 U/mL penicillin-streptomycin (Gibco, Thermo Fisher Scientific).

Prior to RPE differentiations, the hiPSCs lines were tested mycoplasma negative with Venor®GeM Classic (Minerva Biolabs, Berlin, Germany) and thoroughly characterized using previously described methods [13]. Shortly, hiPSC immunofluorescence (IF) labelling for pluripotency markers was done for Nanog (1 : 200, R&D Systems, AF1997), Oct-4 (1 : 200, R&D Systems, AF1759), SSEA-3 (1 : 600, R&D Systems, MAB1434), and Lin-28 (1 : 400, Fisher Scientific, MA1-016), and flow cytometry analyses (BD Accuri C6 Plus)-based quantification for TRA 1–60, SOX2, and Oct3/4. The hiPSC pluripotency was further verified by spontaneous differentiation as EBs, followed by IF labelling for derivative cells of the three embryonic germ layers. To examine chromosomal integrity, the hiPSCs were karyotyped with Giemsa staining (Fimlab Laboratoriot Oy Ltd., Tampere, Finland), and the existence of the heterozygous mutation c.3421C > T, p.Arg1141X was confirmed from the patient fibroblasts and both PXE specific hiPSC lines with BigDye™ Terminator v3.1 Cycle Sequencing (Thermo Fisher Scientific). Further, DNA fingerprinting (STR analysis) of the patient's fibroblasts and the derivative hiPSC lines was performed by Microsynth AG, Switzerland, using highly polymorphic short tandem repeat loci (STRs). STR loci were amplified using the PowerPlex® 16 HS System (Promega). Fragment analysis was done on an ABI3730xl (Life Technologies), and the resulting data were analyzed with GeneMarker HID software (Softgenetics).

### 2.2. RPE Differentiation and Cell Culture

The RPE differentiation and cryopreservation of the RPE cells were initially conducted to both PXE-specific iPSC lines as previously described [12]. For the further molecular and functional assessment, one hiPSC line from the patient (PXE006.TAU.f.D, abbreviation PXE006F here), one from the age/gender-matched healthy control (WT007.TAU.f.A, abbreviation WT007F here), as well as one hESC line (Regea08/017, abbreviation 08/017 used here), was used. Before experiments, cells were seeded with a density of 2.5 ∗ 10^6^ cells/cm^2^ on a porous polyethylene terephthalate (PET) hanging cell culture inserts (0.3 cm^2^, pore size 1.0 *μ*m, Sarstedt) which were coated with collagen IV (10 *μ*g/cm^2^, Sigma) and LN521 (1.8 *μ*g/cm^2^, Biolamina) in PBS (Gibco) containing Mg^2+^ and Ca^2+^. Serum-free culture medium consisting of KO-DMEM, supplemented with 15% KnockOut™ serum replacement, 2 mM Gluta-MAX™ (Gibco), 0.1 mM 2-mercaptoethanol (Gibco), 1% MEM nonessential amino acids (Gibco), and 50 U/ml penicillin-streptomycin, was used. To reach RPE maturity, cells were cultured for 8 to 10 weeks before the characterization experiments [14].

### 2.3. RPE Characterizations

For RPE analysis with IF, the cells were fixed and immunostained as previously described [12]. Primary antibodies ABCC6 (1 : 200, SantaCruz), Ezrin (1 : 200, Abnova), Bestrophin (1 : 200, Abcam), CRALBP (1 : 200, Abcam), Na^+^/K^+^-ATPase (1 : 200, Abcam), Claudin19 (CL19, 1 : 200, R&D), and Zonula occludens 1 (ZO1,1 : 200, Invitrogen) and secondary antibodies goat anti-rat Alexa Fluor 568 (1 : 200, Invitrogen), donkey anti-goat Alexa Fluor 488 (1 : 200, Invitrogen), donkey anti-mouse Alexa Fluor 488 (1 : 200, Invitrogen), and donkey anti-rabbit Alexa Fluor 568 (1 : 200, Invitrogen) were used. A laser scanning confocal microscope (LSM800, Zeiss) with 63x/1.4 oil immersion objective was used to capture Z-stack images from the IF-stained samples. Z-stack images were converted to maximum intensity projections (MIP) with ImageJ [15, 16].

Pigmentation of RPE monolayers was analyzed for all samples at the time point of maturation (8 weeks) after seeding cells on cell culture inserts (*n* = 3 for each cell line). Differential interference contrast (DIC) images were captured with laser scanning confocal microscopy (LSM800, Zeiss, 20x air immersion objective), and quantification was conducted by calculating average brightness from 5-7 randomly selected areas of the monolayer. Thus, a total of 16-17 region of interests (ROIs) per each analyzed cell line was included in the quantification.

Transepithelial electrical resistance (TER) was measured from RPE monolayers on cell culture inserts with a Millicell electrical resistance volt-ohm meter (Merck Millipore). Each measurement was repeated twice, and average TER values were calculated (*n* = 24–26 inserts for each cell line). As a background value, an insert without cells was measured and the value was subtracted from the TER values.

Ca^2+^ imaging of cultured RPE monolayers was conducted as previously described [14, 17]. Briefly, the RPE monolayers were loaded with Ca^2+^-sensitive dye Fluo-4-acetoxymethyl ester (1 mM, Fluo-4 AM; Molecular Probes, Thermo Fisher Scientific) for 45 minutes. Before and after the loading, the cells were washed with Elliot buffer solution (pH 7.3, 330 mOsm). Gravity-fed solution exchange system (AutoMate Scientific) was used during the imaging for the perfusion of the cells with Elliot buffer alone or Elliot containing 100 *μ*M ATP (Sigma Aldrich). Elevated increases in free intracellular Ca^2+^ concentration [Ca^2+^]_I_ was imaged with a Nikon Eclipse FN1 upright fluorescence microscope with a 25x water immersion objective (NA = 1.10). Total imaging time was 10 minutes consisting of 2 minutes of baseline imaging, 2 minutes of ATP stimulus, and 6 minutes of additional imaging. Data analysis was conducted with ImageJ and Matlab (R2018b) from cell culture inserts (*n* = 3 for each cell line) with three randomly selected regions. Each ROI consists of approximately 100 cells which were outlined with ImageJ. The intensity data as a function of time was converted to Matlab form and analyzed with the script package previously developed and described in [17]. Two subcategories were determined: cells that respond to ATP stimulus and cells that do not respond to ATP stimulus. In addition, a relative maximum amplitude of the Ca^2+^ response was calculated from each of the responding cells.

The phagocytosis assay was conducted as previously described [12]. The photoreceptor outer segment (POS) particles (isolated from porcine retinas) were suspended in an RPE medium containing 10% fetal bovine serum (FBS, Gibco) which was added to the apical side of the RPE monolayer and incubated for 2 h at 37°C with 5% CO_2_. The cells were fixed and IF stained. Primary antibody anti-opsin (1 : 1000, Sigma) was used to label POS particles. F-actins were detected with Phalloidin (1 : 800, Sigma) to observe the internalized POS particles. Calculation of the internalized POS particles from inserts (*n* = 2 insert per cell line, each with 5 ROI) is described in [14]. Each ROI consists of approximately 100 cells.

All statistics were performed with Mann–Whitney U with GraphPad Prism test to compare statistical significances. A *p* value of ≤0.05 was considered statistically significant.

## 3. Results

### 3.1. Generation of High-Quality hiPSC Lines from a PXE Patient

**Figure 1:**
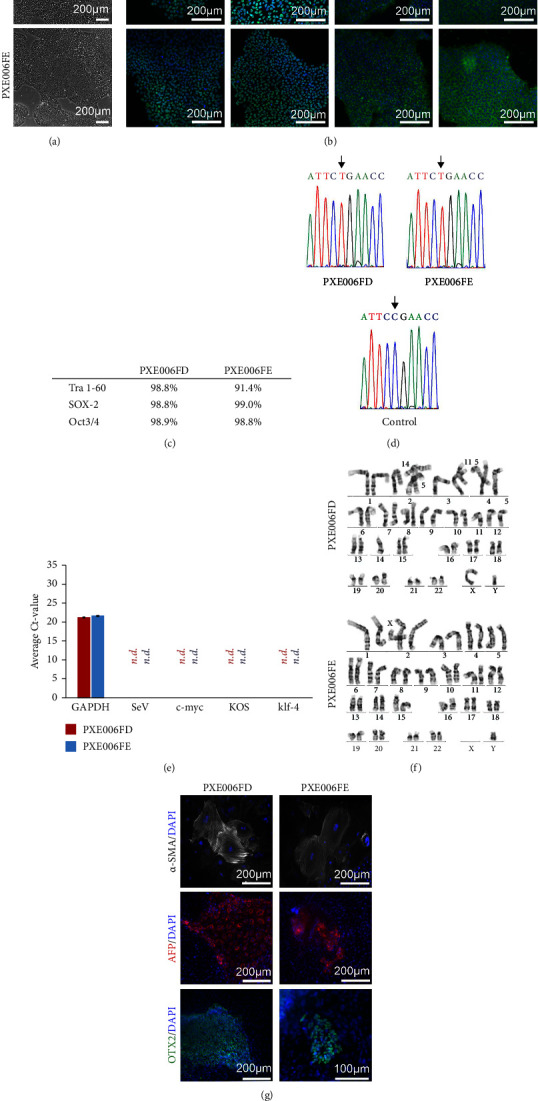
(a) hiPSC colony morphology of PXE patient-specific hiPSC lines (PXE006FD and PXE006FE) by phase contrast microscopy. (b) Expression of pluripotency markers nanog, Oct-4, SSEA-3, and Lin28 in fluorescence microscopy images. Nuclei counterstained with DAPI. (c) Flow cytometry analyses results for pluripotency markers TRA 1–60, SOX-2, and Oct-3/4. (d) The presence of the point mutation (C to T) is shown in the electropherogram analyzed by targeted Sanger sequencing. (e) Removal of the reprogramming factors confirmed with qRT-PCR (n.d. for not detected). Both PXE hiPSC lines showed expression only for the GAPDH housekeeping gene. (f) 46, XY karyogram shown for both PXE hiPSC lines with normal diploid karyotype of 46, XY assed by giemsa banding. (g) *In vitro* pluripotency assessment with shown in fluorescence microscopy images as expression of marker proteins for the three germ layers after EB formation and analyses of differentiation of three germ layers. Scale bar 200 *μ*m and 100 *μ*m.

The two successfully established PXE patient-specific hiPSC lines (PXE006FD and PXE006FE) displayed correct, uniform hiPSC morphology (Figure 1(a)). The pluripotency of the hiPSCs was confirmed by IF staining using antibodies for pluripotency markers Nanog, Oct-4, SSEA-3, and Lin28 (Figure 1(b)). Further flow cytometry analyses for TRA 1–60, SOX-2, and Oct-3/4 showed high (>90%) positivity for these pluripotency markers (Figure 1(c)). The two PXE-specific hiPSC lines were confirmed for the presence of the disease-specific point mutation (C to T) by targeted Sanger sequencing (Figure 1(d)). The successful removal of the exogenously introduced pluripotency reprogramming factors as well as the Sendai virus vector was shown by quantitative RT-PCR (Figure 1(e)). Both PXE-specific hiPSC lines showed normal diploid karyotype of 46, XY as analyzed by Giemsa banding (Figure 1(f)) and were mycoplasma negative (Supplementary Figure S1D). The capability of the hiPSC lines to differentiate *in vitro* into derivative cells of the three embryonic germ layers: mesoderm, ectoderm, and endoderm by spontaneous embryoid body (EB) differentiation followed by IF staining for alpha-smooth muscle actin (*α*-SMA, mesoderm marker), orthodenticle homeobox-2 (OTX2, ectoderm marker), and alpha-fetoprotein (AFP, endoderm marker) (Figure 1(g)) was successfully confirmed. Genetic identity was shown by STR profiling against the parental dermal fibroblasts. Electropherograms are shown in Supplementary Figure S1A-C, and the full STR report is available with the authors. Thus, both established PXE-specific hiPSC lines demonstrated high-quality and full pluripotency characteristics important for further use of the established cell lines.

### 3.2. RPE-Specific Marker Expression Analyses and Quantification of the Cell Pigmentation Reveals Differences in PXE-Specific RPE as Compared to the Controls

**Figure 2:**
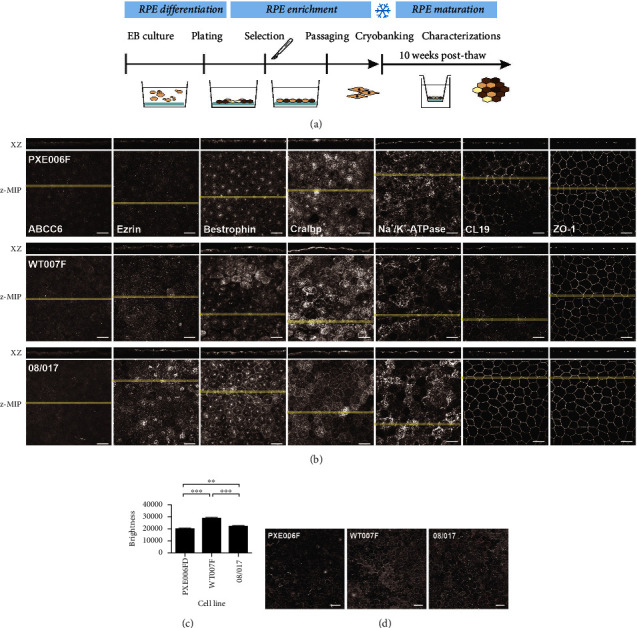
(a) The schematic view of the RPE differentiation process. (b) Immunofluorescence images of cultured RPE cells at the time point of 10 weeks. Each presented image consists of a laser scanning confocal microscopy z-maximum intensity projection (z-MIP) and xz cross section (MIP from 20 sections). Scale bar 20 *μ*m. (c) Pigmentation analysis of RPE monolayers (^∗∗^*p* ≤ 0.01 and ^∗∗∗^*p* ≤ 0.001) cultured for 8 weeks. The brightness was calculated from five differential interference contrast (DIC) images from each cell line. (d) Representative single DIC images of RPE monolayer cultures were used for quantification where higher brightness indicates lower pigmentation. Scale bar 20 *μ*m. Statistics performed with Mann–Whitney U test. Data represents mean ± SEM.

After the RPE differentiation process (Figure 2(a)) with previously established methods, both PXE-specific hiPSC lines differentiated towards RPE cells with morphological and pigmentation characteristics (Supplementary Figures S3a and S3c). However, when RPE cells were cultured on inserts, clear rearrangement of actin fibers was visible in the 006FE cell line (Supplementary Figure S3b); thus, the 006FE cell line was discarded from the additional analyses. In the further analyses using one of the lines, no difference in the differentiation efficacy was detected with the PXE hiPSC line (PXE006FD) as compared to the controls (control hiPSC from a healthy donor (WT007F) and human embryonic stem cell line (hESC 08/017) (data not shown). Thus, a sufficient amount of PXE-specific RPE cells could be produced for further molecular and functional assessment.

First, the expression of *ABCC6* gene encoded transporter protein ABCC6 was confirmed with IF labelling in the PXE specific as well as control RPE, and no differences in expression and intracellular location (apical and basal) of the protein were found (Figure 2(b), first column). Next, the expression and intracellular location of the well-acknowledged RPE markers Ezrin, Bestrophin, CRALBP, Na^+^/K^+^-ATPase, CL19, and ZO1 was analyzed, and the data demonstrated similar RPE characteristics of PXE-specific RPE as compared to the controls, except for CL19 and Bestrophin (Figure 2(b), third and sixth columns from left). The intracellular location of the CL19 was more diffuse and cytoplasmic in PXE-specific and healthy hiPSC control (WT007F) as compared to RPE differentiated from the hESC line (08/017), although apical expression was also evident in both. In addition, the expression of Bestrophin seemed to be more heterogeneous across the RPE monolayer in WT007F in comparison with the other cell lines used in this study. Yet, the location of the Bestrophin is still mostly apical in all the cell lines. Representative confocal sections with orthogonal projections after indirect IF labelling are presented for each protein marker in Supplementary Figure S2. Based on the pigmentation quantification of the RPE, the PXE-specific RPE demonstrated a clear (*p* ≤ 0.01) reduction of the brightness and thus increased pigmentation level as compared to health hiPSC (WT007F) and hESC (08/017) controls (Figures 2(c), 2(d) and Supplementary Figure S3c).

### 3.3. Functional Assessment of the Differentiated RPE Reveals Reduced Functionality of PXE-Specific RPE as Compared to the Controls

**Figure 3:**
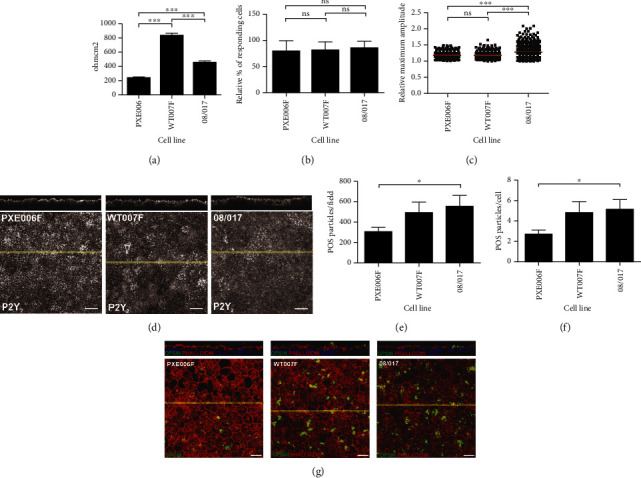
Functional analysis of cultured RPE cells at culture age of 10 weeks. (a) TER values of RPE monolayers. Data represents means ± SEM, ^∗∗∗^*p* ≤ 0.001. Ca^2+^ signaling properties were analyzed with (b) relative % of responding cells and (c) relative maximum amplitudes, ^∗∗∗^*p* ≤ 0.001. In addition, the localization of the P2Y_2_ receptor is shown in (d) as laser scanning confocal microscopy z-MIP and xz cross-sectional images. Scale bar 20 *μ*m. Phagocytosis of RPE cells as internalized POS particles (^∗^*p* ≤ 0.05) per (e) field and (f) cell. (g) Representative laser scanning confocal microscopy z-MIP and xz cross sections. z-MIP images represent the overall number of POS particles in the field. Scale bar 20 *μ*m. All statistics performed with Mann–Whitney U test. Data represent means ± SEM.

The functionality of the differentiated RPE was further analyzed with the panel of functionality assessment methods. First, the TER analyses of the epithelium integrity demonstrated a statistically significant (*p* < 0.001) reduction of the epithelium barrier properties by the PXE-specific RPE as compared to the controls, and interestingly, the healthy control (WT007F) showed even higher TER values as compared to the hESC control (Figure 3(a) and Supplementary Figure S3d).

Next, the Ca^2+^ signaling properties of the RPE were analyzed. We and others have previously shown that examining Ca^2+^ signaling properties is a sensitive method for evaluating the quality and functionality of human pluripotent stem cell (hPSC)-RPE [14, 18]. Here, Ca^2+^ signaling was analyzed by calculating the relative amount of the responding cells (Figure 3(b)) and maximum response amplitudes (Figure 3(c)). Data indicated that there were no significant differences in the intracellular Ca^2+^ activity of PXE-specific RPE as compared to the controls. Instead, both the PXE-specific and the healthy control (WT007F) RPE had significantly lower maximum amplitudes compared to the control hESC RPE (*p* ≤ 0.001) indicating weaker response capability. As the addition of the ATP to the apical side of RPE induces an intracellular Ca^2+^ transient primarily via apical P2Y_2_ receptors [19], the intracellular location of the receptor was analyzed with IF staining, confirming that there was no difference between the compared samples (Figure 3(d)). Last but not least, the important phagocytosis activity of the POS was analyzed. The number of internalized POS particles identified by antiopsin was counted from xz confocal images. Compared to both controls, the PXE-specific RPE demonstrated reduced POS intake efficiency (*p* ≤ 0.05 as compared to 08/017), and there were no differences between the two control RPE (Figures 3(e), 3(f), 3(g)).

## 4. Discussion

The genetic basis of PXE is the inactivation or partial inactivation of the *ABCC6* gene and a spectrum of mutations in the human *ABCC6* gene has been shown to be responsible for PXE [20–24]. The ABCC6 protein is a membrane transporter, for which the substrate remains unknown [24, 25]. ABCC6 protein is primarily expressed at the basolateral plasma membranes of hepatocytes in the liver [26, 27] with lower expression levels in the proximal tubules of the kidney and PXE-affected tissues, including the skin, retina, and blood vessels [20, 28]. However, the reported expression of the protein also in neurons and leukocytes, suggests a more complex multifunctional role with variable intracellular location as well [29, 30].

Regarding retinal complications, previous clinical investigations have shown that retinal angioid streaks occur in 80–95% of PXE patients [31–33]. These streaks are caused by calcification and fracture of the elastic lamina of BM with changes in the outer retina, RPE, and choriocapillaris [34, 35]. These patients are usually asymptomatic until their vision is impaired by complications, including CNV or, less frequently, traumatic BM rupture [36]. The fractures in this membrane result in CNV from the choriocapillaries, and the newly formed fragile blood vessels may break, leading to hemorrhage and scarring. These pathological changes can ultimately lead to progressive loss of visual acuity and, rarely, to legal blindness. In addition to clinical studies, animal models with *ABCC6* mutations including mouse and zebrafish have been established recapitulating part of the PXE manifestations [29, 37]. However, a detailed assessment of the retinal changes has not been performed and the retina-specific disease mechanism remains unknown.

In this study, we aimed to establish an efficient hiPSC-based in vitro disease model to study closer the possible RPE-related mechanisms in PXE-specific human cells. For the hiPSC reprogramming, we used skin fibroblasts isolated from a PXE patient carrying the most common homozygous mutation c.3421C > T, p.Arg1141X on the *ABCC6* gene coding ABCC6 protein [38]. As a result, we were able to establish two distinct hiPSC lines carrying the patient-specific mutation and demonstrating genomic integrity and pluripotency characteristics important for the high-quality hiPSC lines. Thus, the hiPSC reprogramming was successful from the patient's skin fibroblast regardless of the disease phenotype in the skin. Further, based on our existing differentiation methods and RPE maturation cultures [12, 39], we were able to differentiate RPE cells from both established PXE-specific hiPSC lines, although the iPSC line with higher RPE quality was mainly used in downstream analyses.

At the molecular level, ATP-dependent transporters have a major known role in drug delivery in human RPE. In our previous studies, we have already demonstrated that the mature hESC-RPE cells with cobblestone morphology express the *ABCC6* gene coding for the ABCC6 protein [40]. Here, we further confirmed that the PXE-specific RPE as well as control RPEs expressed the transporter protein with similar, mostly apical intracellular localization, although expression was also evident at the basolateral side. Interestingly, it has been previously demonstrated with liver cells that ABCC6 protein has basal localization with Na^+^/K^+^-ATPase transporter [41], and similarly in our study with RPE cells, both transporters share clear apical localization. Thus, further functional assessment would be needed to study if the functionality of the expressed protein due to the mutation is compromised in PXE-specific RPEs. For the establishment of these assays, a better understanding of the functionality of this protein in RPE cells would be important. Previously, Beck et al. [29] have proposed that transport of free radical scavengers, such as reduced glutathione conjugates by ABCC6, could play a critical role in ensuring an appropriate concentration of antioxidants in the extracellular matrix (ECM), thus ensuring the correct assembly and deposition of ECM polymers such as elastic fibers. It remains to be studied if similar functionality exists in RPE cells and thus having a possible role in BM elastic lamina calcification mediated through RPE basal lamina as has been previously proposed for the aging BM and macula [42].

In the retina, the RPE has multiple functions: absorption of light energy, transport of metabolites and nutrients between photoreceptors and choriocapillaris, expression of growth factors for photoreceptors, regulation of homeostasis of the ionic environment, phagocytosis of the POS, regulation of visual cycle, and creation of the blood-retinal barrier [43]. Here, we aimed to further characterize the expression of some of the important proteins involved in these processes as well as key functionalities important for the healthy RPE. Our analyses indicated that the PXE-specific differentiated RPE cells from both used iPSC lines were highly pigmented as compared to healthy controls, and together with RPE-specific protein characterizations, this may indicate proper differentiation and maturation level of PXE-specific RPE. Whether the detected higher pigmentation of PXE-specific RPE *in vitro* has any clinical relevance remains to be studied further, and no specific conclusion can be made with these preliminary findings using only 2 iPSC lines established from one PXE patient.

Our IF analyses also demonstrated the similar expression and intracellular location of the RPE markers Ezrin, Bestrophin, CRALBP, and Na^+^/K^+^-ATPase as compared to the healthy controls. Interestingly, the tight junction barrier–related protein CL19 responsible for the formation of the tight junction barrier in RPE [44] showed clearly more diffuse and cytoplasmic location in PXE-specific (and also in healthy control RPE) as compared to hESC-differentiated RPE. In addition, the TER measurements further showed a statistically significant reduction of the epithelium barrier properties by the PXE-specific RPE as compared to the controls. On the other hand, the expression of ZO1, a tight junction–associated protein, did not show a similar trend. Interestingly, Liu et al. have demonstrated that CL19 knockdown has a similar effect with reduced TER and ZO1 remaining associated with apical junctional complex [44]. Also, the knockdown of CL19 has also been shown to affect phagocytosis efficacy [44], and similarly in our recent study, the most pronounced reductions in CL19 junctional localization and phagocytosis activity were correlated [39]. Here, a similar trend is seen as the phagocytosis assay showed a clearly reduced POS intake efficiency by PXE-specific RPE as compared to the controls. The malfunctions in phagocytosis in general, can lead to accumulation of POS particles and lipofuscin in RPE. Interestingly, it has been also reported that some of the PXE patients have an accumulation of lipofuscin in some areas of the retina [4, 45]. Thus, the possible role of reduced phagocytosis intake activity or POS degradation in PXE pathogenesis serves further investigations in the future.

The purinergic signaling is important for the integrative functions of the retina and RPE. Intact ATP signaling can be considered as one key indicator of the RPE functionality. With our previously developed analysis tools for Ca^2+^ imaging [17], the PXE-specific RPE cells demonstrated the ability to respond to the ATP stimulus, but the response amplitude was weaker as compared to the control cells although the expression levels of the P2Y2 receptor were equal in all compared samples. We have previously shown that the higher RPE maturation level increases the response amplitudes [14]; thus, further studies are needed to conclude if the PXE-specific RPE cells were more immature (although highly pigmented) with the phenotype or if the *ABCC6* mutation has some still unknown function in the barrier properties, phagocytosis or ATP signaling responses of RPE.

In this study, two hiPSC lines were generated from a single PXE patient; thus, all findings are preliminary in nature and the number of patients is a limitation of the study. New hiPSC lines should be established from additional PXE patients for future analyses and the validation of the potential RPE-related findings reported here. This is especially important for the potential individual effects that may cause bias in interpretations of this clinically highly variable disease phenotype. In the future, methods such as differentiation of retinal organoids from the PXE-specific hiPSC lines provide additional tools to study retinal-specific disease mechanisms at a retinal organoid level as well as novel tools to establish additional PXE-specific in vitro disease models for other affected tissues including skin and arterial tissues via differentiation of tissue-specific cells from hiPSCs [46, 47].

## 5. Conclusions

As a conclusion, we were able to establish two PXE patient-specific hiPSC lines with good pluripotency characteristics and the study confirmed feasible RPE differentiation using these cell lines. The ability to differentiate RPE from these hiPSCs offers a renewable disease-specific cell source for in vitro studies. The molecular and functional assessment of the PXE-specific RPE demonstrated increased RPE pigmentation and reduced epithelial barrier functions as well as phagocytosis activity as compared to healthy controls, indicating possible local RPE-dependent factors that might explain the individual vulnerability of the retinas for macular degeneration in PXE. The established cell models are available for further PXE-specific disease modelling and for the validation of the interesting preliminary research findings with additional PXE patients.

## Data Availability

The datasets generated during and/or analyzed during the current study are available from the corresponding author upon request.

## Supplementary Materials

Supplementary Materials Figure S1: electropherograms of the STR profiling and results of the mycoplasma analyses; Figure S2: expression and subcellular localization of marker protein; Figure S3: (a) phase contrast micrographs of PXE-specific hiPSC lines PXE006FD and PXE006FE, healthy control line WT007F, and hESC line 08/017. Scale bar 100 *μ*m. (b) Basolateral localization of actin cytoskeleton. Phalloidin was used to stain the actin cytoskeleton. Scale bar 20 *μ*m. (c) Pigmentation analysis of all the cell lines used in this study. (*n* = 3 inserts from each cell line, 5–7 ROIs per insert) (^∗∗^*p* ≤ 0.01 and ^∗∗∗^*p* ≤ 0.001). (d) TER values of RPE monolayers. TER measured from 24–26 inserts from PXE006FD, WT007F, and 08/017. 10 inserts measured from PXE006FE. Data represent means ± SEM (^∗∗∗^*p* ≤ 0.001).

## References

[1] Li Q., van de Wetering K., Uitto J. (2019). Pseudoxanthoma elasticum as a paradigm of heritable ectopic mineralization disorders: pathomechanisms and treatment development. *American Journal Of Pathology*.

[2] Jansen R. S., Duijst S., Mahakena S. (2014). ABCC6-mediated ATP secretion by the liver is the main source of the mineralization inhibitor inorganic pyrophosphate in the systemic circulation-brief report. *Arteriosclerosis, Thrombosis, and Vascular Biology*.

[3] Kozák E., Bartstra J. W., de Jong P. A. (2023). Plasma level of pyrophosphate is low in pseudoxanthoma elasticum owing to mutations in the ABCC6 gene, but it does not correlate with ABCC6 genotype. *Journal of Clinical Medicine*.

[4] Risseeuw S., Bennink E., Poirot M. G. (2020). A reflectivity measure to quantify Bruch’s membrane calcification in patients with pseudoxanthoma elasticum using optical coherence tomography. *Translational Vision Science & Technology*.

[5] Booij J. C., Baas D. C., Beisekeeva J., Gorgels T. G. M. F., Bergen A. A. B. (2010). The dynamic nature of Bruch’s membrane. *Progress in Retinal and Eye Research*.

[6] Väärämäk S., Uusitalo H., Tőkési N., Pelttari S., Váradi A., Nevalainen P. I. (2019). Pyrophosphate treatment in pseudoxanthoma elasticum (PXE)-Preventing ReOcclusion after surgery for critical limb ischaemia. *Surgical Case Reports*.

[7] Risseeuw S., Ossewaarde-van Norel J., Klaver C. C. W., Colijn J. M., Imhof S. M., van Leeuwen R. (2019). Visual acuity in pseudoxanthoma elasticum. *Retina*.

[8] Finger R. P., Issa P. C., Schmitz-Valckenberg S., Holz F. G., Scholl H. N. (2011). Long-term effectiveness of intravitreal bevacizumab for choroidal neovascularization secondary to angioid streaks in pseudoxanthoma elasticum. *Retina*.

[9] Gliem M., Müller P. L., Birtel J. (2017). Quantitative fundus autofluorescence in pseudoxanthoma elasticum. *Investigative Ophthalmology & Visual Science*.

[10] Hess K., Gliem M., Birtel J. (2020). Impaired dark adaptation associated with A diseased Bruch membrane in pseudoxanthoma elasticum. *Retina*.

[11] Hess K., Gliem M., Charbel Issa P. (2020). Mesopic and scotopic light sensitivity and its microstructural correlates in pseudoxanthoma elasticum. *JAMA Ophthalmology*.

[12] Hongisto H., Ilmarinen T., Vattulainen M., Mikhailova A., Skottman H. (2017). Xeno- and feeder-free differentiation of human pluripotent stem cells to two distinct ocular epithelial cell types using simple modifications of one method. *Stem Cell Research & Therapy*.

[13] Hongisto H., Dewing J. M., Christensen D. R. (2020). In vitro stem cell modelling demonstrates a proof-of-concept for excess functional mutant TIMP3 as the cause of Sorsby fundus dystrophy. *The Journal of Pathology*.

[14] Viheriälä T., Sorvari J., Ihalainen T. O. (2021). Culture surface protein coatings affect the barrier properties and calcium signalling of hESC-RPE. *Scientific Reports*.

[15] Schindelin J., Arganda-Carreras I., Frise E. (2012). Fiji: an open-source platform for biological-image analysis. *Nature Methods*.

[16] Schneider C. A., Rasband W. S., Eliceiri K. W. (2012). NIH Image to ImageJ: 25 years of image analysis. *Nature Methods*.

[17] Sorvari J., Viheriälä T., Ilmarinen T., Ihalainen T. O., Nymark S. (2019). Analysis of ATP-induced Ca2+ responses at single cell level in retinal pigment epithelium monolayers. *Advances in Experimental Medicine and Biology*.

[18] Miyagishima K. J., Wan Q., Corneo B. (2016). In pursuit of authenticity: induced pluripotent stem cell-derived retinal pigment epithelium for clinical applications. *Stem Cells Translational Medicine*.

[19] Mitchell C. H., Reigada D. (2008). Purinergic signalling in the subretinal space: a role in the communication between the retina and the RPE. *Purinergic Signalling*.

[20] Bergen A. A., Plomp A. S., Schuurman E. J. (2000). Mutations in ABCC6 cause pseudoxanthoma elasticum. *Nature Genetics*.

[21] Ringpfeil F., Lebwohl M. G., Christiano A. M., Uitto J. (2000). Pseudoxanthoma elasticum: mutations in the MRP6 gene encoding a transmembrane ATP-binding cassette (ABC) transporter. *Proceedings of the National Academy of Sciences of the United States of America*.

[22] Meloni I., Rubegni P., De Aloe G. (2001). Pseudoxanthoma elasticum: point mutations in the ABCC6 gene and a large deletion including also ABCC1 and MYH11. *Human Mutation*.

[23] Struk B., Cai L., Zäch S. (2000). Mutations of the gene encoding the transmembrane transporter protein ABC-C6 cause pseudoxanthoma elasticum. *Journal of Molecular Medicine*.

[24] Armentano M. F., Ostuni A., Infantino V., Iacobazzi V., Castiglione Morelli M. A., Bisaccia F. (2008). Identification of a new splice variant of the human ABCC6 transporter. *Research Letters in Biochemistry*.

[25] Váradi A., Szabó Z., Pomozi V., de Boussac H., Fülöp K., Arányi T. (2011). ABCC6 as a target in pseudoxanthoma elasticum. *Current Drug Targets*.

[26] Madon J., Hagenbuch B., Landmann L., Meier P. J., Stieger B. (2000). Transport function and hepatocellular localization of mrp6 in rat liver. *Molecular Pharmacology*.

[27] Scheffer G. L., Hu X., Pijnenborg A. C. L. M., Wijnholds J., Bergen A. A. B., Scheper R. J. (2002). MRP6 (ABCC6) detection in normal human tissues and tumors. *Laboratory Investigation*.

[28] Kool M., van der Linden M., de Haas M., Baas F., Borst P. (1999). Expression of human MRP6, a homologue of the multidrug resistance protein gene MRP1, in tissues and cancer cells. *Cancer Research*.

[29] Beck K., Hayashi K., Nishiguchi B., Saux O. L., Hayashi M., Boyd C. D. (2003). The distribution of Abcc6 in normal mouse tissues suggests multiple functions for this ABC transporter. *Journal of Histochemistry and Cytochemistry*.

[30] Beck K., Hayashi K., Dang K., Hayashi M., Boyd C. D. (2005). Analysis of ABCC6 (MRP6) in normal human tissues. *Histochemistry and Cell Biology*.

[31] Janotka H., Hess J., Włodarczyk J. (1995). [Angioid streaks. Pathogenesis and the clinical picture]. *Klinika Oczna*.

[32] Gurwood A. S., Mastrangelo D. L. (1997). Understanding angioid streaks. *Journal of the American Optometric Association*.

[33] Pelttari S., Väärämäki S., Vanakker O. (2022). Various vascular malformations are prevalent in Finnish pseudoxanthoma elasticum (PXE) patients: a national registry study. *Orphanet Journal of Rare Diseases*.

[34] Hansen M. S., Klefter O. N., Larsen M. (2014). Retinal degeneration and persistent serous detachment in the absence of active choroidal neovascularization in pseudoxanthoma elasticum. *Acta Ophthalmologica*.

[35] Gliem M., Müller P. L., Birtel J., Hendig D., Holz F. G., Charbel Issa P. (2016). Frequency, phenotypic characteristics and progression of atrophy associated with a diseased Bruch’s membrane in pseudoxanthoma elasticum. *Investigative Ophthalmology & Visual Science*.

[36] Georgalas I., Papaconstantinou D., Koutsandrea C. (2009). Angioid streaks, clinical course, complications, and current therapeutic management. *Therapeutics and Clinical Risk Management*.

[37] Sun J., She P., Liu X., Gao B., Jin D., Zhong T. P. (2020). Disruption of Abcc6 transporter in zebrafish causes ocular calcification and cardiac fibrosis. *International Journal of Molecular Sciences*.

[38] Pfendner E. G., Vanakker O. M., Terry S. F. (2007). Mutation detection in the ABCC6 gene and genotype-phenotype analysis in a large international case series affected by pseudoxanthoma elasticum. *Journal of Medical Genetics*.

[39] Viheriälä T., Hongisto H., Sorvari J., Skottman H., Nymark S., Ilmarinen T. (2022). Cell maturation influences the ability of hESC-RPE to tolerate cellular stress. *Stem Cell Research & Therapy*.

[40] Juuti-Uusitalo K., Vaajasaari H., Ryhänen T. (2012). Efflux protein expression in human stem cell-derived retinal pigment epithelial cells. *PLoS One*.

[41] Pomozi V., Le Saux O., Brampton C. (2013). ABCC6 is a basolateral plasma membrane protein. *Circulation Research*.

[42] Pilgrim M. G., Lengyel I., Lanzirotti A. (2017). Subretinal pigment epithelial deposition of drusen components including hydroxyapatite in a primary cell culture model. *Investigative Ophthalmology & Visual Science*.

[43] Steinberg R. H. (1985). Interactions between the retinal pigment epithelium and the neural retina. *Documenta Ophthalmologica*.

[44] Liu F., Xu T., Peng S., Adelman R. A., Rizzolo L. J. (2020). Claudins regulate gene and protein expression of the retinal pigment epithelium independent of their association with tight junctions. *Experimental Eye Research*.

[45] Shiraki K., Kohno T., Moriwaki M., Yanagihara N. (2001). Fundus autofluorescence in patients with pseudoxanthoma elasticum. *International Ophthalmology*.

[46] Sharma A., Sances S., Workman M. J., Svendsen C. N. (2020). Multi-lineage human iPSC-derived platforms for disease modeling and drug discovery. *Cell Stem Cell*.

[47] Sharma K., Krohne T. U., Busskamp V. (2020). The rise of retinal organoids for vision research. *International Journal of Molecular Sciences*.

