# Supplementary material for: Novel Human Induced Pluripotent Stem Cell-Based Model for Retinal Pigment Epithelial Cells to Reveal Possible Disease Mechanisms for Macular Degeneration in Pseudoxanthoma Elasticum
Source: J Ophthalmol. 2024 Sep 21;2024:6939920. doi: 10.1155/2024/6939920 (PMC11438508; doi:10.1155/2024/6939920)
Supplement: Supplementary Materials — Figure S1: electropherograms of the STR profiling and results of the mycoplasma analyses; Figure S2: expression and subcellular localization of marker protein; Figure S3: (a) phase contrast micrographs of PXE-specific hiPSC lines PXE006FD and PXE006FE, healthy control line WT007F, and hESC line 08/017. Scale bar 100 μm. (b) Basolateral localization of actin cytoskeleton. Phalloidin was used to stain the actin cytoskeleton. Scale bar 20 μm. (c) Pigmentation analysis of all the cell lines used in this study. (n = 3 inserts from each cell line, 5–7 ROIs per insert) (∗∗p ≤ 0.01 and ∗∗∗p ≤ 0.001). (d) TER values of RPE monolayers. TER measured from 24–26 inserts from PXE006FD, WT007F, and 08/017. 10 inserts measured from PXE006FE. Data represent means ± SEM (∗∗∗p ≤ 0.001). [file 6939920.f1.docx]

**Supplementary Materials**

**
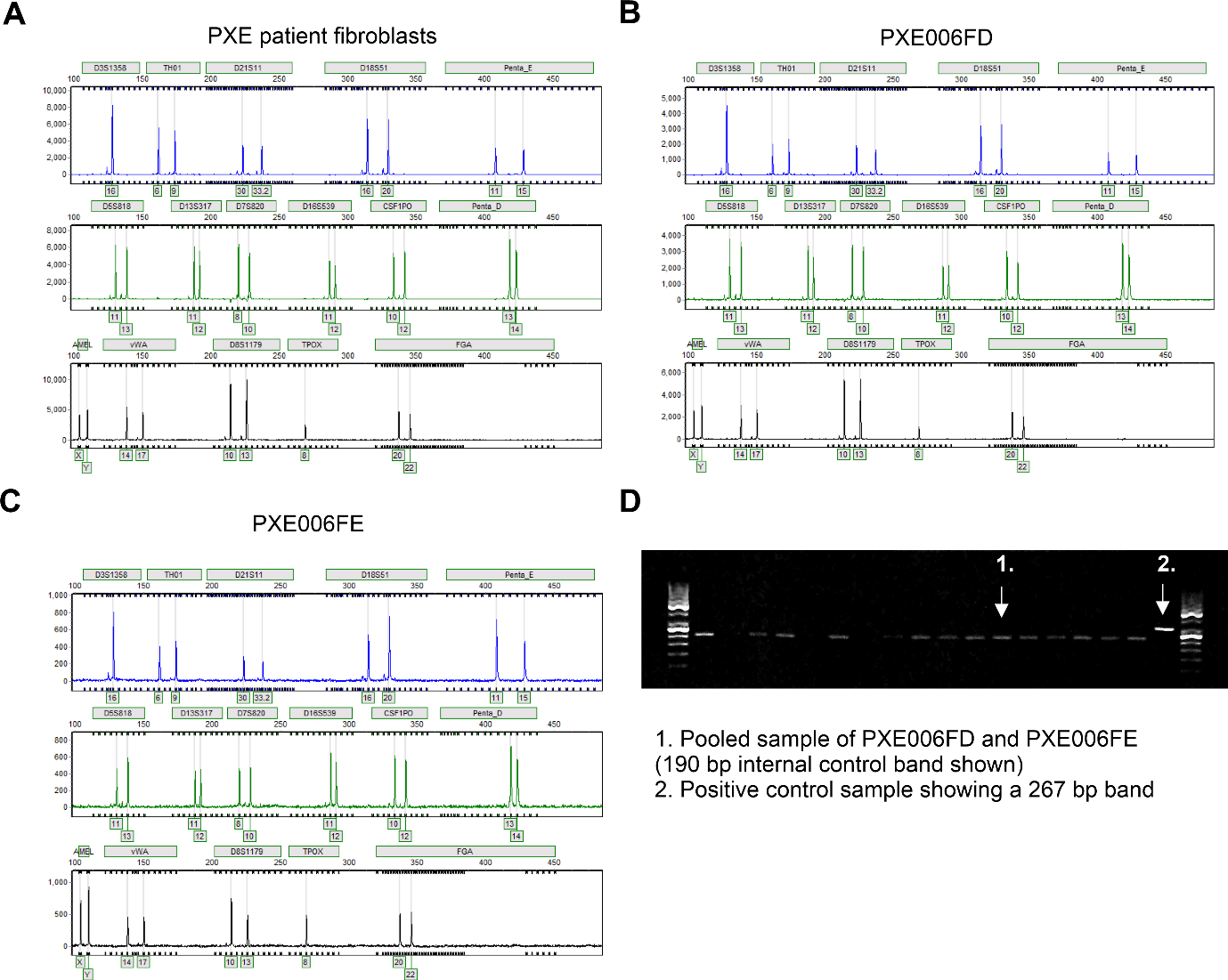
**

**Figure S1:** Electropherograms of the STR profiling and results of the mycoplasma analyses. Genetic identity of both of the established PXE specific hiPSC lines PXE006FD (**B**) and PXE006FE (**C**) was shown by STR profiling to match the parental dermal fibroblasts (**A**). Both established hiPSC lines were mycoplasma negative (**D**).


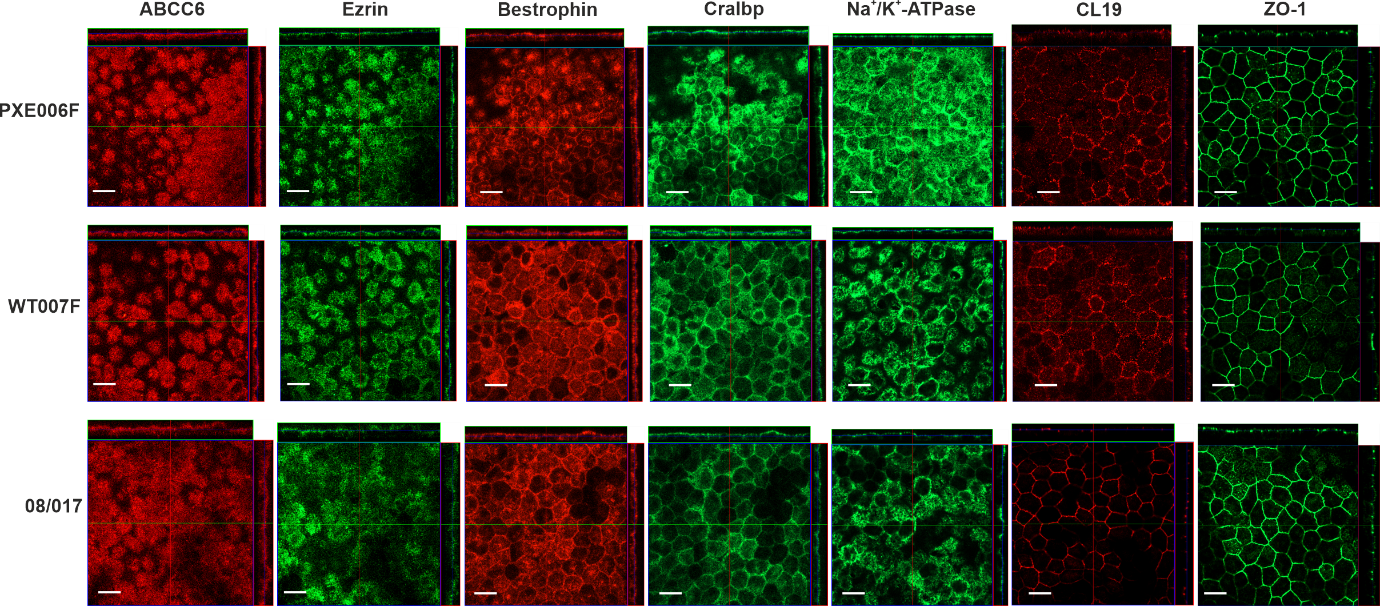


**Figure S2:** Expression and subcellular localization of marker proteins. Representative confocal sections with orthogonal projections after indirect immunofluorescence labeling with PXE-associated ABCC6, microvilli-associated Ezrin, visual cycle protein CRALBP, ion channels bestrophin and Na+/K+-ATPase, and tight junction proteins CL19 and ZO-1. Scale bars 20 µm.


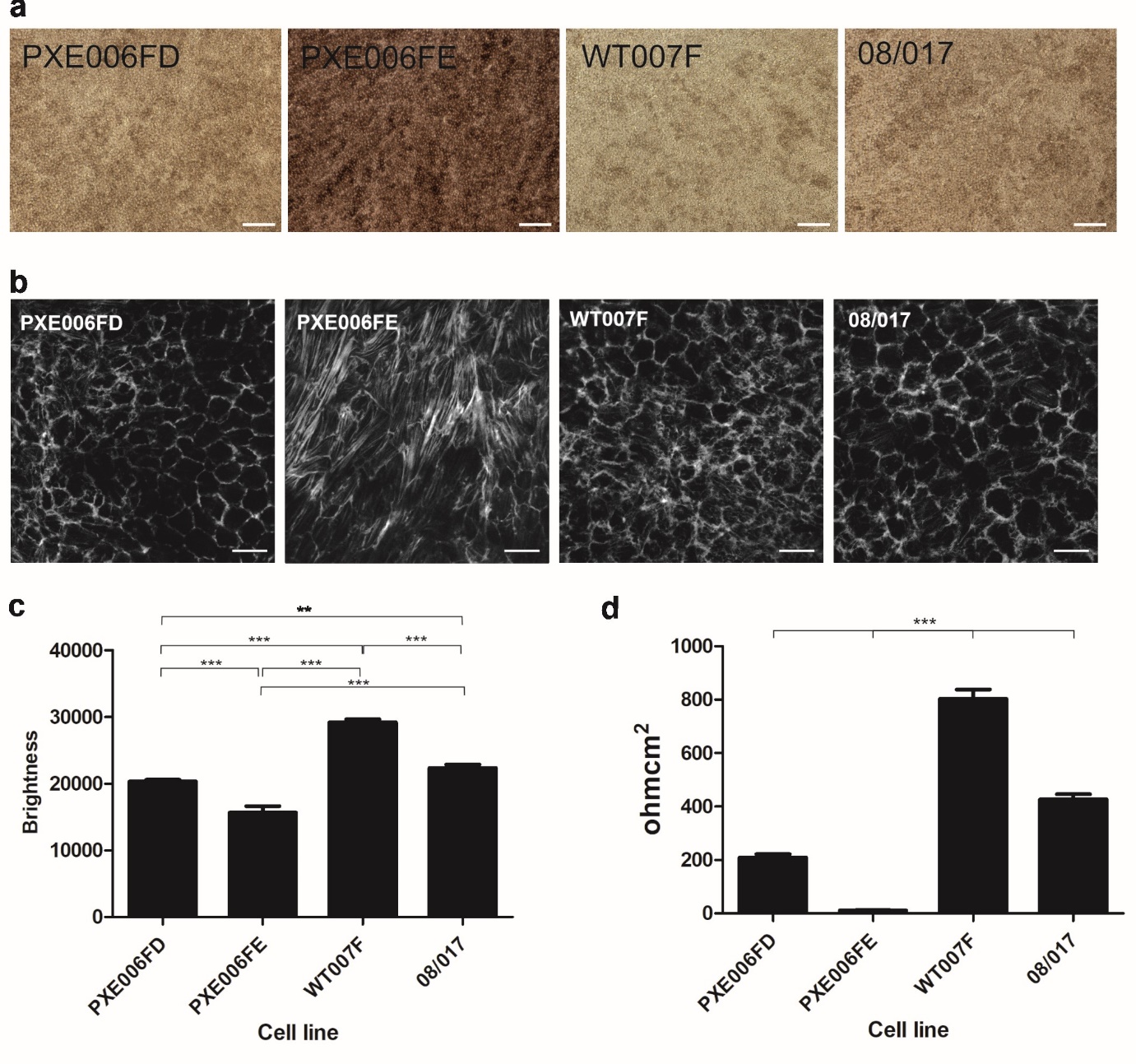


**Figure S3: a)** Phase contrast micrographs of PXE-specific hiPSC lines PXE006FD and PXE006FE, healthy control line WT007F, and hESC line 08/017. Scale bar 100 µm. **b)** Basolateral localization of actin cytoskeleton. Phalloidin was used to stain the actin cytoskeleton. Scale bar 20 µm. **c)** Pigmentation analysis of all the cell lines used in this study. (n=3 inserts from each cell line, 5-7 ROIs per insert) (** p≤0.01, *** p≤0.001). **d)** TER values of RPE monolayers. TER measured from 24-26 inserts from PXE006FD, WT007F, and 08/017. 10 inserts measured from PXE006FE. Data represents means ±SEM (*** p≤0.001).
